# New generation VMAT2 inhibitors induced parkinsonism

**DOI:** 10.1016/j.prdoa.2020.100078

**Published:** 2020-11-07

**Authors:** Rani Priyanka Vasireddy, Brent Sokola, Zain Guduru

**Affiliations:** a University of Kentucky Medical Center, United States

## Abstract

• Drug induced parkinsonism caused by valbenazine and deutetrabenazine.
• Possible side effects of VMAT-2 inhibitors.
• Valbenazine and deutetrabenazine can unmask underlying Parkinson’s Disease.

## 1 Introduction

Tardive syndrome (TS), is one of the most common drug-induced movement disorders. TS is primarily caused by the prolonged use of dopamine-blocking agents such as antipsychotics. Tetrabenazine is vesicular monoamine transporter type 2 (VMAT-2) inhibitor which is FDA approved for chorea in Huntington’s disease and also used off-label for TS. VMAT-2 inhibitors deplete monoamines such as dopamine, norepinephrine, serotonin, and histamine and are expected to reduce dyskinetic movements by reducing dopamine neurotransmission. However, tetrabenazine use is currently discouraged due to its side effect profile that includes depression and parkinsonism [1]. In 2017, valbenazine and deutetrabenazine were FDA approved for the treatment of tardive syndrome. Valbenazine and deutetrabenazine are VMAT-2 inhibitors and derivatives of tetrabenazine designed to increase the half-life and decrease fluctuations in plasma levels. These pharmacokinetic changes were targeted in hopes to reduce the frequency of adverse effects while providing consistent movement control.

In phase 3 randomized, double-blind, placebo-controlled trials for both valbenazine and deutetrabenazine, the side effect profile was limited primarily to somnolence, akathisia, and dry mouth, with no reports of drug induced parkinsonism (DIP) from valbenazine in the KINECT 3 trial, one patient identified with DIP in the KINECT 3 extension (Factor et al. (2017). *J Clin Psychiatry*), and only one case of DIP from deutetrabenazine the AIM-TS trial [2], [3]. In this paper, we report two cases of VMAT2 inhibitor induced DIP: one associated with valbenazine and one with deutetrabenazine.

## 2 Case 1

A 59-year-old man treated for depression with valproic acid for about 10 years was being switched to aripiprazole (15 mg/day) in 2015. On examination in 2017, he had only stereotypical movements involving tongue, mouth, face and head regions, cervical dystonia and blepharospasm (AIMS-TD score 12). Aripiprazole was stopped within a month of onset of TS symptoms. In May of 2018, deutetrabenazine was started for TS and titrated to 21 mg twice a day. Within a few weeks, the TS symptoms had improved, but the patient complained of somnolence and dizziness. These effects persisted even after reduction of the dose to 12 mg twice a day. Tapering further to 6 mg qAM and 12 mg qPM resolved the somnolence and dizziness, but TS reappeared. Deutetrabenazine was changed to valbenazine 80 mg daily. With this dose, he had somnolence and therefore, the dose was decreased to 40 mg daily. In the next 2 months of initiation of this medication, TS symptoms significantly improved but the patient presented with right hand resting tremor and slow walking, and on examination he had asymmetrical parkinsonism (MDS-UPDRS III score: 18). Patient preferred to continue valbenazine at 40 mg/day despite the Parkinsonian symptoms for his TS. With slow progression of parkinsonism, carbidopa/levodopa 25/100 mg 1 tab by mouth three times a day was added, and the patient initially reported significant improvement in his parkinsonism and continued to have improvement in TS. On follow-up, his tremor had returned and his DAT scan showed decreased uptake in his right putamen. Please see the attached DAT scan image below.

## 3 Case 2

An 86-year-old woman with gastroparesis who was on metoclopramide for twenty years presented with involuntary tongue movements for 6 months. Metoclopramide was immediately stopped, and the involuntary movements worsened to involve the trunk too. On examination in August of 2019, she had oro-bucco-lingual movements and truncal dyskinetic movements s/o TS (AIMS-TD score 14). She was started on deutetrabenazine and the dose was titrated to 18 mg twice a day. In the next 6 weeks, TS was significantly improved but she complained of slowness of spontaneous movements, along with a masked face and low-pitched voice. On examination in October 2019, she was noted to have symmetrical parkinsonism (bradykinesia-rigidity predominant) and the dose of deutetrabenazine was decreased to 12 mg twice a day. Within a few weeks, parkinsonism has resolved and continued to have significant improvement of TS. Patient refused a DAT scan.

## 4 Discussion

We report two cases of VMAT-2 inhibitors induced Parkinsonism of which one of them is a man in whom we propose that the underlying Parkinson’s disease (based on decreased uptake in right putamen and clinical presentation) was unmasked secondary to newly introduced dopamine-depleting medication. In case 1, we added carbidopa/levodopa to manage DIP symptoms as the patient was experiencing a significant benefit from valbenazine. In Case 2, we still consider one of the two possibilities of either unmasking the underlying Parkinson’s disease (more likely) or a Drug induced Parkinsonism secondary to deutetrabenazine as we couldn’t get a DAT scan for more definitive answer. To access the video of both the patients exhibiting signs and symptoms of Parkinsonism, click on the image below (online version only).

Based on the review of literature to-date there has been only one article that addressed valbenazine-induced parkinsonism, our cases demonstrate a similar side effect profile [4]. There are no comparator trials between these medications and this adverse effect is relatively rare so it is not advised to compare the results of these clinical trials for prevalence or severity. Per the prescribing information, the frequency of Parkinsonism for valbenazine is 3% and less than 1% in those who received placebo. One of 221 patients treated with deutetrabenazine in the AIM-TS trial was reported to develop Parkinsonism. With tetrabenazine, 10% prevalence of Parkinsonism was reported in 48 week open label study. Valbenazine and deutetrabenazine are considered as first line choices of pharmacotherapy for TS [5] but are not devoid of unmasking of underlying PD or DIP. The clinicians should be cautious and maintain a high index of clinical suspicion for DIP, in individuals treated with newer generation VMAT2 inhibitors, irrespective of the dose.

## Declaration of Competing Interest

The authors declare that they have no known competing financial interests or personal relationships that could have appeared to influence the work reported in this paper.
