# Supplementary material for: New generation VMAT2 inhibitors induced parkinsonism
Source: Clin Park Relat Disord. 2020 Nov 7;3:100078. doi: 10.1016/j.prdoa.2020.100078 (PMC8298827; doi:10.1016/j.prdoa.2020.100078)
Supplement: Supplementary data 1 [file mmc1.docx]

**Video Captions**

Patient 1: Valbenazine unmasking underlying Parkinson’s disease.

1A) Before initiation of valbenazine: Tardive dyskinesia: Abnormal, involuntary stereotypical movements noted in tongue, mouth, face and head regions. These movements are better with distraction. No bradykinesia. 1B) After initiation of valbenazine: Asymmetrical Parkinsonism: Right hand resting tremor of 5 hz frequency and low amplitude. Right more than left bradykinesia. No tardive dyskinetic movements are noted. He persists to have significant improvement of TD.

Patient 2: Deutetrabenazine induced parkinsonism vs unmasking underlying Parkinson’s disease.

2A) Before initiation of deutetrabenazine: Tardive dyskinesia: Abnormal, involuntary stereotypical movements noted in jaw, tongue, oral and truncal regions. In addition, she has laryngeal dyskinesia. 2B) One month after initiation of deutetrabenazine: She has significant improvement of TD. No signs of parkinsonism are noted. 2C) Symmetrical parkinsonism: Persists to have significant improvement of TD. Symmetrical bradykinesia, masked face, hypophonia, absent arm swing, stooped posture, shuffling gait.
