# Supplementary figures and images for: New generation VMAT2 inhibitors induced parkinsonism
Source: Clin Park Relat Disord. 2020 Nov 7;3:100078. doi: 10.1016/j.prdoa.2020.100078 (PMC8298827; doi:10.1016/j.prdoa.2020.100078)

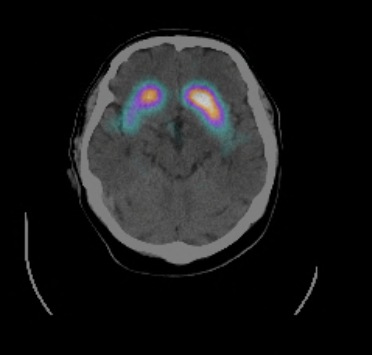

Supplement: Supplementary data 2 [file mmc2.zip › DAT scan image.PNG]
